# Vertical-Injection AlGaInP LEDs with n-AlGaInP Nanopillars Fabricated by Self-Assembled ITO-Based Nanodots

**DOI:** 10.1186/s11671-015-1064-3

**Published:** 2015-09-15

**Authors:** Ho-Soung Ryu, Min Joo Park, Seung Kyu Oh, Hwa-Sub Oh, Jong-Hyeob Baek, Joon Seop Kwak

**Affiliations:** Department of Printed Electronics Engineering, Sunchon National University, Jeonnam, 540-742 South Korea; LED Device Research Center, Korea Photonics Technology Institute, Gwangju, 500-779 South Korea

**Keywords:** AlGaInP, Light-emitting diodes, Nanopillars, Nanodots, 78.67.-n, 78.67.De, 78.67.Qa

## Abstract

The light output power of AlGaInP-based vertical-injection light-emitting diodes (VI-LEDs) can be enhanced significantly using n-AlGaInP nanopillars. n-AlGaInP nanopillars, ~200 nm in diameter, were produced using SiO_2_ nanopillars as an etching mask, which were fabricated from self-assembled tin-doped indium oxide (ITO)-based nanodots formed by the wet etching of as-deposited ITO films. The AlGaInP-based VI-LEDs with the n-AlGaInP nanopillars provided 25 % light output power enhancement compared to VI-LEDs with a surface-roughened n-AlGaInP because of the reduced total internal reflection by the nanopillars at the n-AlGaInP/air interface with a large refractive index difference of 1.9.

## Background

Given the recent strong interest in the epitaxial quality of AlGaInP-based materials, the application of AlGaInP-based light-emitting diodes (LEDs) has been extended to automotive lighting, full-color displays, and visible light communications, which require high-brightness and high-power operation [1–4]. A vertical-injection geometry through wafer-bonded Si conductive substrates with a reflective electrode has been suggested as a solution to increase the light output power of AlGaInP-based LEDs because vertical-injection LEDs (VI-LEDs) yield better current spreading, good heat dissipation, and simple packaging [5, 6]. On the other hand, the light extraction efficiency (LEE) of AlGaInP VI-LEDs is limited by the total internal reflection because of the large difference in refractive index between the n-AlGaInP (*n* ∼ 2.9) and air (*n* = 1.0) [7].

Several beneficial methods that can enhance the LEE of AlGaInP LEDs have been reported. AlGaInP LEDs with a roughened surface, textured surface, and truncated pyramid geometry were reported to enhance the LEE by increasing the critical angle and the probability of escape of emitted light from an air/semiconductor interface [8–10]. Omni-directional reflectors as a p-type electrode and an air-hybrid distributed Bragg reflector structure were also reported to improve the LEE of AlGaInP LEDs significantly [11, 12]. Recently, Wenjing et al. suggested that the fabrication of AlGaInP-based nanorod LEDs using self-assembly metal layer nanomasks can enhance the probability of emitted light escaping from nanorod LEDs [13]. Although the nanorods fabricated using Au metal clusters as nanomasks improved the LEE of AlInGaP LEDs greatly by enhancing the probability of escape of emitted light, this approach cannot be implemented in high-power AlGaInP-based VI-LEDs with wafer-bonded Si conductive substrates, because the formation of Au metal clusters requires high temperature annealing (>400 °C), which is significantly higher than that of wafer bonding between the Si conductive wafer and AlGaInP LED wafer.

The present study focused on improving the LEE of high-power AlGaInP-based VI-LEDs with wafer-bonded Si conductive substrates. For this purpose, n-AlGaInP nanopillars with a diameter of ~200 nm were fabricated using self-assembled ITO-based nanodots as etching nanomasks. This approach is a promising method for producing high-power AlGaInP-based VI-LEDs with wafer-bonded Si conductive substrates because ITO-based nanodots can be produced by the wet etching of as-deposited ITO films without an annealing process. The results show that the light output power of AlGaInP-based VI-LEDs with n-AlGaInP nanopillars can be improved considerably compared to that of the VI-LEDs with surface-roughened n-AlGaInP, which is a widely implemented method for increasing the LEE of AlGaInP-based VI-LEDs.

## Methods

AlGaInP-based LEDs emitting a 610-nm wavelength were grown on 2-in. (100) GaAs substrates by metal-organic vapor phase epitaxy (MOVPE). The AlGaInP-based LED structure consisted of a GaInP etching stop layer and an n-GaAs contact layer grown on an n-GaAs buffer layer, a 2-μm-thick Si-doped n-AlGaInP cladding layer, an undoped active layer with 20 period AlGaInP/GaInP multiple quantum wells (MQWs), a Mg-doped p-AlGaInP layer, and a thick p^+^-GaP window layer.

To fabricate the AlGaInP-based VI-LEDs with a chip size of 1 mm^2^, Ni/Ag/Ni reflectors were deposited onto the p^+^-GaP contact layer and annealed at 350 °C for 1 min to ensure ohmic contact. Adhesive/barrier/bonding layers consisting of Ni/Cr/Ni/Au/Sn/Au were then deposited onto the Ni/Ag/Ni reflectors, and the AlGaInP-based LEDs on the GaAs substrates were bonded to the p-Si conductive substrates at 350 °C for 1 min in a nitrogen environment. After wafer bonding, the GaAs substrate and GaInP etching stop layer were removed using a NH_4_OH-based chemical etching solution. The Ni/Ge/Au contacts were then deposited on the n-GaAs contact layer, and Ti/Au boding pad metals were deposited on n-AlGaInP and the back side of the p-Si conducting substrate. Finally, n-AlGaInP nanopillars, ~200 nm in diameter, were produced using SiO_2_ nanopillars as an etching mask, which were fabricated by self-assembled ITO-based nanodots formed by wet etching of the as-deposited ITO films. For comparison, surface-roughened n-AlGaInP was also fabricated by wet etching of the n-AlGaInP layer using a H_3_PO_4_-based solution.

After fabricating the AlGaInP-based VI-LEDs with n-AlGaInP nanopillars or surface-roughened n-AlGaInP, the wafers were diced and the AlGaInP-based VI-LED chips were mounted onto the TO-18 headers with no epoxy encapsulation. All subsequent measurements were carried out using a conventional integration sphere. The n-AlGaInP nanopillars and ITO-based nanodots were examined by scanning electron microscopy (SEM).

## Results and Discussion

**Fig. 1:**
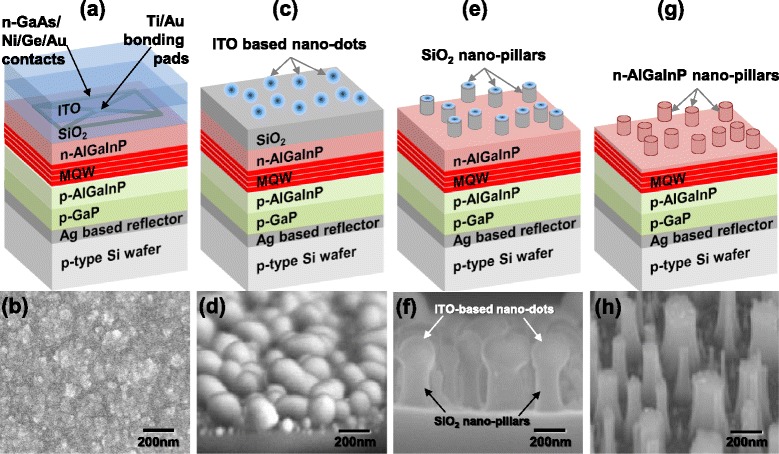
**a**, **c**, **e**, **g** Schematic diagrams of the fabrication process for n-AlGaInP nanopillars. **b**, **d**, **f**, **h** SEM images for each fabrication step

Figure 1 presents schematic diagrams of the fabrication process for the n-AlGaInP nanopillars and SEM images for each fabrication step. After depositing the ITO layer on top of the SiO_2_ layer, as shown in Fig. 1a, b, the ITO layer yielded a rough surface and low transparency, suggesting that an oxygen-poor metallic phase was dominant at the as-deposited ITO layer by electron-beam evaporation. The VI-LED samples with the ITO layer were dipped into a chemical solution containing HCl, which resulted in the formation of nanodots from the ITO films, as shown in Fig. 1c, d. The mean diameter of the ITO-based nanodots was 200 nm. The formation of self-assembled ITO-based nanodots by wet etching of the as-deposited ITO films can be explained by the formation of a small crystalline ITO phase imbedded in a metallic amorphous matrix in the as-deposited ITO films, followed by the selective etching of a metallic amorphous matrix during wet etching [14]. After the formation of the self-assembled ITO-based nanodots, reactive ion etching was then performed to produce SiO_2_ nanopillars using the ITO-based nanodots as an etching mask, which transferred the island pattern of the nanodots to the SiO_2_, as shown in Fig. 1e, f. Finally, to produce the n-AlGaInP nanopillars, the n-AlGaInP layer was etched by inductively coupled plasma (ICP) using the SiO_2_ nanopillars as an etching mask, as shown in Fig. 1g, h. ICP etching of the n-AlGaInP was performed using a HBr and Ar gas mixture at a flow rate of 24 and 12 sccm, respectively. The residual SiO_2_ nanopillar mask was removed by a buffered oxide etchant solution.

**Fig. 2:**
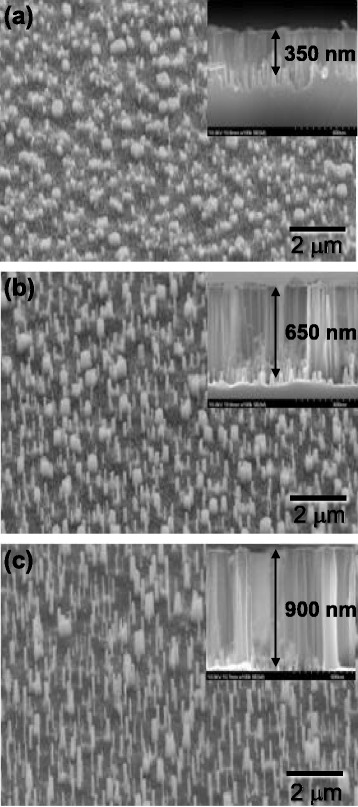
SEM images of the n-AlGaInP nanopillars fabricated with a process time for ICP etching of **a** 40, **b** 60, and **c** 80 s

**Fig. 3:**
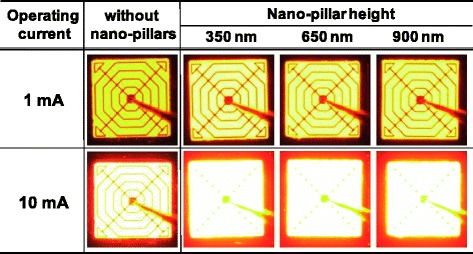
Light emission images at an operating current of 1 and 10 mA for the AlGaInP-based VI-LEDs with/without the n-AlGaInP nanopillars with different heights

In this study, to examine the effects of the height of the n-AlGaInP nanopillars on the output power of the AlGaInP-based VI-LEDs, the processing time for the ICP etching of the n-AlGaInP was varied from 40 to 80 s, and as shown in Fig. 2, the height of the n-AlGaInP nanopillars was varied from 350 to 900 nm by increasing the ICP etch time. The diameter and density of the n-AlGaInP nanopillars were not changed significantly as the ICP etch time was increased, indicating that ICP etching with a HBr and Ar gas mixture did not etch the sidewalls of the nanopillars significantly. Figure 3 shows the light emission images at an operating current of 1 and 10 mA for the AlGaInP-based VI-LEDs with/without the n-AlGaInP nanopillars with different heights. The light emission images of the AlGaInP-based VI-LEDs with the n-AlGaInP nanopillars were much brighter than those of the VI-LEDs without nanopillars at both 1 and 10 mA. This suggests that the n-AlGaInP nanopillars can enhance the LEE of the AlGaInP-based VI-LEDs. In addition, the height of the n-AlGaInP nanopillars has little influence on the light emission images of the AlGaInP-based VI-LEDs, as shown in Fig. 3, indicating that the n-AlGaInP nanopillars with a height of 350 nm are insufficient to increase the probability of emitted light escaping from the air/semiconductor interface.

**Fig. 4:**
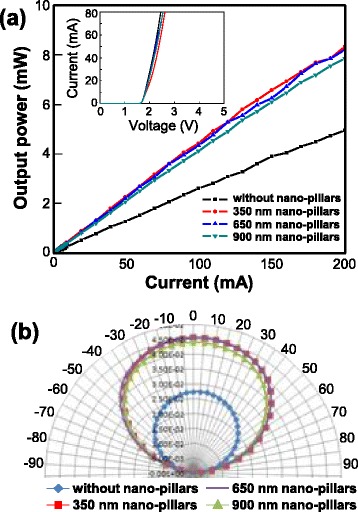
**a** Variation of the light output power as a function of the applied current for the AlGaInP-based VI-LEDs with/without the n-AlGaInP nanopillars having different heights. The *inset* shows the current-voltage curves of the AlGaInP-based VI-LEDs with/without the nanopillars with different heights. **b** Far-field emission patterns for the VI-LEDs with/without the nanopillars with different heights

Figure 4a shows the variation of the light output power as a function of the applied current for the AlGaInP-based VI-LEDs with/without the n-AlGaInP nanopillars with different heights. The output power of the AlGaInP-based VI-LEDs with nanopillars with a height of 350, 650, and 900 nm was 4.1, 4.0, and 3.8 mW at 80 mA, respectively. In contrast, that of the VI-LEDs without the nanopillars was only 2.3 mW at 80 mA, which clearly shows that the nanopillars can improve the LEE of the VI-LEDs considerably. The far-field emission patterns of the VI-LEDs with/without the nanopillars, as shown in Fig. 4b, confirmed the improvement of the LEE for the VI-LEDs with the nanopillars. The emission angle of the VI-LEDs with the nanopillars (129°) was wider than that of the VI-LEDs without the nanopillars (124°). In addition, the emission intensity of the VI-LEDs with the nanopillars was much higher than that of the VI-LEDs without the nanopillars at emission angles ranging from −90° to 90°. The AlGaInP-based VI-LEDs with/without the nanopillars yielded a marginal difference in the operating voltage, as shown in the inset of Fig. 4a. This suggests that the formation of the n-AlGaInP nanopillars using the SiO_2_ nanopillars as an etch mask during the ICP etch process caused little damage to the VI-LEDs. This clearly shows that the LEE of the AlGaInP VI-LEDs was improved by the formation of n-AlGaInP nanopillars.

**Fig. 5:**
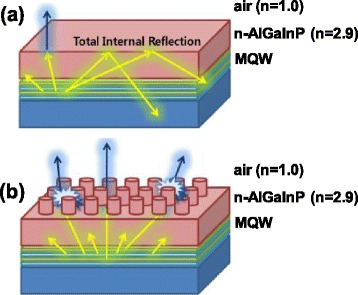
**a** Schematic diagrams of the AlGaInP-based VI-LEDs without the n-AlGaInP nanopillars. **b** Schematic diagrams of the AlGaInP-based VI-LEDs with the n-AlGaInP nanopillars, which shows the reduced total internal reflection by the nanopillars

The improvement in the output power of the AlGaInP-based VI-LEDs with the nanopillars can be explained as follows. As shown in Fig. 5a, the LEE of AlGaInP VI-LEDs without the nanopillars is limited by the large difference in refractive index between the n-AlGaInP (*n* ∼ 2.9) and air (*n* = 1.0), resulting in a small critical angle of ~20° for the total internal reflection from a flat surface according to Snell’s law, followed by only 11 % extraction of input power [15]. On the other hand, as shown in Fig. 5b, the LEE of the AlGaInP VI-LEDs with the n-AlGaInP nanopillars was enhanced significantly because the nanopillars can increase the critical angle and the probability of photons escaping from the semiconductor-to-air interface [16].

**Fig. 6:**
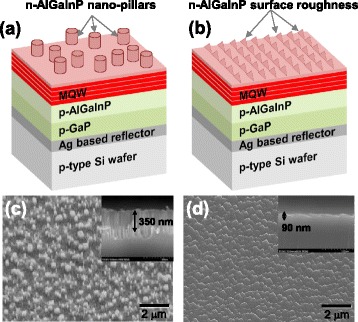
Schematic diagrams and SEM images of **a, c** the AlGaInP-based VI-LEDs with the n-AlGaInP nanopillars and **b, d** the VI-LEDs with the surface-roughened n-AlGaInP

**Fig. 7:**
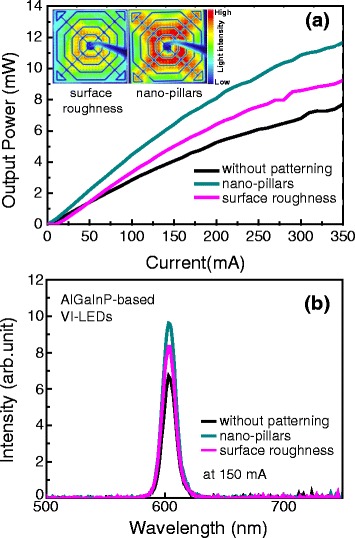
**a** Variation of the output power as a function of current for the AlGaInP-based VI-LEDs with n-AlGaInP nanopillars, for the VI-LEDS with the surface-roughened n-AlGaInP, and for VI-LEDs without patterning. The *inset* shows the emission profile at 50 mA for the VI-LEDs with nanopillars and for the VI-LEDs with the surface-roughened n-AlGaInP. **b** EL spectra for the VI-LEDs with n-AlGaInP nanopillars, for the VI-LEDS with the surface-roughened n-AlGaInP, and for VI-LEDs without patterning

Finally, light output power of the AlGaInP-based VI-LEDs with n-AlGaInP nanopillars was compared with that of the VI-LEDs with the surface-roughened n-AlGaInP, which is a widely implemented method to increase the LEE of the AlGaInP-based VI-LEDs. For this purpose, AlGaInP-based VI-LEDs with n-AlGaInP nanopillars as well as the VI-LEDs with the surface-roughened n-AlGaInP were fabricated, as shown in Fig. 6. The n-AlGaInP nanopillars with a height of 350 nm were fabricated using the same procedure shown in Fig. 6a, c. The surface-roughened n-AlGaInP was produced by wet etching of the n-AlGaInP layer using the HCl- and H_3_PO_4_-based solution for 400 s. As shown in Fig. 6b, d, a triangle-like morphology roughness with a height of ~90 nm was produced, which was tilted towards a specific direction. This is believed to be related to the surface polarity of the n-AlGaInPs [17, 18]. Figure 7a shows the variation of the light output power as a function of the applied current for the AlGaInP-based VI-LEDs with the n-AlGaInP nanopillars and the VI-LEDS with the surface-roughened n-AlGaInP. For comparison, the light output power of the AlGaInP-based VI-LEDs without patterning is also shown. As presented in Fig. 7a, the AlGaInP-based VI-LEDs with the surface-roughened n-AlGaInP yielded 20 % light output power enhancement at 350 mA compared to the VI-LEDs without patterning, due to the reduced total internal reflection by the triangle-like morphology roughness at the n-AlGaInP/air interface. On the other hand, the output power of the VI-LEDs with the n-AlGaInP nanopillars was 11.5 mW at 350 mA, which is a 25 % improvement in output power compared to the VI-LEDs with the surface-roughened n-AlGaInP. This can be attributed to the height of the n-AlGaInP nanopillars (~350 nm) being four times higher than that of the triangle-like-roughened n-AlGaInP (~90 nm), followed by an increase in the probability of escape for the light emitted from an air/semiconductor interface. The height of the nanopillars is close to half of the emitted wavelength, which can further enhance the extraction of the light generated at the MQWs. The light intensity in the emission profile at 50 mA for the VI-LEDs with the n-AlGaInP nanopillars was much higher than that for the VI-LEDs with the surface-roughened n-AlGaInP, as shown in the inset of Fig. 7a. Furthermore, the electroluminescence (EL) spectra at 150 mA for the VI-LEDs with the n-AlGaInP nanopillars revealed a higher EL intensity than that of the VI-LEDs with the surface-roughened n-AlGaInP, as shown in Fig. 7b. This strongly suggests that the LEE of the AlGaInP-based LEDs can be improved further using the n-AlGaInP nanopillars compared to that of the AlGaInP-based LEDs with the conventional surface-roughened n-AlGaInP.

## Conclusions

This paper reports an improvement of the LEE of the AlGaInP-based VI-LEDs with wafer-bonded Si conductive substrates using the n-AlGaInP nanopillars. Nanopillars with a diameter of ~200 nm were produced using SiO_2_ nanopillars as an etching mask, which were fabricated by self-assembled ITO-based nanodots formed by the wet etching of as-deposited ITO films without an annealing process. The height of the n-AlGaInP nanopillars were varied from 350 to 900 nm, and n-AlGaInP nanopillars with a height of 350 nm were sufficient to increase in the probability of emitted light escaping from the air/semiconductor interface. The AlGaInP-based VI-LEDs with the n-AlGaInP nanopillars provided 25 % light output power enhancement compared to the VI-LEDs with the surface-roughened n-AlGaInP because the height of the n-AlGaInP nanopillars was four times higher than that of the triangle-like-roughened n-AlGaInP.
